# Prioritization preferences for COVID-19 vaccination are consistent across five countries

**DOI:** 10.1057/s41599-022-01392-1

**Published:** 2022-12-07

**Authors:** Simon Munzert, Sebastian Ramirez-Ruiz, Başak Çalı, Lukas F. Stoetzer, Anita Gohdes, Will Lowe

**Affiliations:** 1 Hertie School, Berlin, Germany; 2 University Witten/Herdecke, Witten, Germany

**Keywords:** Science, technology and society, Social policy

## Abstract

Vaccination against COVID-19 is making progress globally, but vaccine doses remain a rare commodity in many parts of the world. New virus variants require vaccines to be updated, hampering the availability of effective vaccines. Policymakers have defined criteria to regulate who gets priority access to the vaccination, such as age, health complications, or those who hold system-relevant jobs. But how does the public think about vaccine allocation? To explore those preferences, we surveyed respondents in Brazil, Germany, Italy, Poland, and the United States from September to December of 2020 using ranking and forced-choice tasks. We find that public preferences are consistent with expert guidelines prioritizing health-care workers and people with medical preconditions. However, the public also considers those signing up early for vaccination and citizens of the country to be more deserving than later-comers and non-citizens. These results hold across measures, countries, and socio-demographic subgroups.

## Introduction

Researchers, governments, and the pharmaceutical industry took on the development of a vaccine against the coronavirus disease 2019 (COVID-19) in an unprecedented effort. By the end of 2021, a majority of countries had approved the use of at least one vaccine, and around seven billion doses had been administered globally (Mathieu et al., 2021).^1^ The distribution of these doses, however, remains concentrated in a privileged set of countries, ensuring that global access remains a challenge (Forman et al., 2021; Wouters et al., 2021). Given initial and ongoing supply limitations, a key challenge to the distribution of COVID-19 vaccines has been optimizing access. Because not everyone can be vaccinated immediately, national policymakers have had the responsibility, in addition to infrastructure and logistics, to develop access priority and eligibility criteria.

In this article, we study public preferences over who deserves priority access to COVID-19 vaccination. Public support for vaccination prioritization policies matters both for ensuring the effective cooperation of all individuals in curbing the current and future pandemics through vaccination and to overcome resistance towards public health campaigns. While public opinion does not determine vaccination prioritization criteria, policy-making is more effective when it understands and responds to gaps between public preferences and policies. Moreover, even when public preferences are aligned with policies, this information helps demonstrate the legitimacy of decisions in contexts where elite responsiveness to public demand has been questioned (Flores et al., 2022; Green et al., 2020).

Against this backdrop, we designed a survey that was fielded in Brazil, Germany, Italy, Poland, and the US between 8 September and 9 December 2020 (*n*_Total_ = 4366), eliciting prioritization preferences through a ranking task and a conjoint experiment. In the first task, respondents had to rank eight groups according to which group should get vaccinated sooner or later. The groups varied on traits such as age, profession, and health status, but also included various elite groups (political, economic, and sports), allowing us to compare principles corresponding with official prioritization guidelines with less common ones. In the second task, we implement a forced-choice conjoint experiment, asking respondents to decide which of two fictional persons with randomly varied attributes should be prioritized for access to the vaccine. Attributes included gender, age, having children, occupation, citizenship, pre-existing medical condition, and early registration for vaccination. This allowed us to isolate individual attribute effects and assess how they contribute to decisions where there are concrete trade-offs.

We find that despite varying ethical principles and government guidelines about which groups should be prioritized for access to the COVID-19 vaccine, *public* preferences are remarkably consistent across the five countries. The cross-national evidence shows that the public consistently prioritizes workers in the health-care system, people with medical preconditions, and the elderly. Perhaps surprisingly, there is little variation in preferences across countries or respondent characteristics. However, we also find that the public is willing to discriminate according to other criteria, including citizenship status and vaccine registration timing following the first-come, first-served principle. The first highlights the challenges of inclusive public health measures, not merely as a matter of social solidarity but also public safety and efficient service provision. The second points to the importance of fairness considerations in rolling out vaccination programs during health emergencies.

Our findings complement evidence from previous studies on vaccine prioritization preferences (Ceccato et al., 2021; Duch et al., 2021; Gollust et al., 2020; Knotz et al., 2021a; Luyten et al., 2022; Persad et al., 2021; Reeskens et al., 2021; Schaeffer and Haderup Larsen, 2022), by highlighting that the public’s prioritization preferences are not only remarkably consistent *across* the five countries we study, but also *within* each country, and suggesting low levels of polarization about who should be prioritized for COVID-19 vaccination.

## Principles and practices of COVID-19 vaccination prioritization

The responsibility for establishing guidelines for the distribution of COVID-19 vaccines has fallen mostly to individual countries.^2^ In the initial stages of the pandemic, national mandates targeted the highest priority groups (older people, health-care workers with risk of contact, and patients and personnel of long-term care facilities). However, countries differed in their categorization of those most at-risk. For example, the US prioritized essential frontline non-healthcare workers (Dooling et al., 2021). Brazil included vulnerable social and ethnic groups, e.g., indigenous and Quilombola communities (Ministério da Saúde, 2021). And Italy included educational, prison staff, law enforcement, and armed forces regardless of their health status and age (Ministero della Salute, 2021). Table 1 summarizes the differences and commonalities for the countries we study in our analysis.

**Table 1:** Overview of initial national vaccination priority allocation ordinances in countries of study (as of December 2020).

	BRA	DEU	ITA	POL	USA
Age	x	x	x	x	x
Medical preconditions	x	x	x	x	x
Early registration					
Citizenship					
Gender					
Children					
Contact with high-risk		x	x		
*Occupation*					
Health-care workers	x	x	x	x	x
Education	x	x	x	x	
Infrastructure	x	x			
Social services	x	x	x	x	x
State functioning		x			
Public order	x	x	x	x	
Essential*					x
Non-healthcare frontline^a^					x
Marginalized groups	x				

^a^This criterion can include various occupations.

Existing practice by authorities largely reflects a utilitarian perspective to vaccine prioritization. Societal harm is minimized by targeting those who would be at greatest risk of harm from the disease without being vaccinated (Persad et al., 2020), e.g., the elderly and those with pre-existing conditions, as these attributes increase the likelihood of severe consequences from an infection. Also consistent with a utilitarian perspective would be to favor people whose loss due to illness would also carry larger societal consequences and who also face a higher risk of getting infected, e.g., frontline workers. These are primarily workers in the health-care system, but also those with high exposure to the public. If the public were to follow the utilitarian heuristic, we would expect to see support for prioritization of the elderly, health-care workers and others who face an increased risk of infection due to social contact, and of those with medical preconditions.

Vaccine prioritization can also be motivated with the “deservingness” heuristic, often used in research on social welfare (Petersen et al., 2012), migration (De Coninck and Matthijs, 2020), and health care (Jensen and Petersen, 2017). High “deservingness” accrues to those in objective need of the resource, who have no control over their circumstances, or are deserving on the basis of past behavior. Applied to vaccine prioritization, Knotz et al. (2021a, p. 299) argue that health-care workers should be considered the most deserving, followed by those with pre-existing conditions, the elderly, and others with increased risk exposure, such as teachers or police officers. In that sense, applying the deservingness heuristic would lead to outcomes similar to the utilitarian heuristic. However, public understanding of deservingness might also be influenced by shared social identities Reeskens and Van der Meer (2019), such as partisanship (Stoetzer et al., 2022) or co-citizenship (Helbling et al., 2022; Knotz et al., 2021b), which would be in conflict with standard ethical guidelines. We would expect participants following this logic to sympathize with members of their in-group. In that context, it would also be interesting to study the perceived deservingness of elite groups, for example politicians and athletes, for which a large part of the population is unlikely to feel a strong common identity.

Obviously, the public might care little about classical perspectives and employ other norms and heuristics instead. For instance, given the popularity of the “women and children first” principle, it is imaginable that prioritizing women (vs. men) and children (vs. the elderly) in the context of COVID-19 vaccination is a popular strategy in the public, even though the practical relevance of the rule in face of disaster seems overblown (Elinder and Erixson, 2012). While we do not have any expectations for whether women and children will be prioritized, there might be plausible reasons to see them as more deserving. Women may be prioritized because of the importance and risks of care work in society, which women take on to a greater extent in society. In doing so, they expose themselves to higher infection risks, which in turn might result in shortages in care work. Children, on the other hand, are usually perceived to be particularly worthy of protection, and could be considered the more effective target group (Giubilini et al., 2021).

## Existing evidence

Our central question is how public preferences on COVID-19 vaccination prioritization are structured within and across country contexts. Evidence on that question is rapidly accumulating. One set of studies focuses on global distribution preferences, asking whether countries from the Global South vs. Global North (or low vs. high-income countries) should be prioritized (Clarke et al., 2021; Guidry et al., 2021; Klumpp et al., 2022; Steinert et al., 2022; Vanhuysse et al., 2021). Most of those studies across different contexts find a majority of the public to support the donation of vaccines to low-income countries, but also within-country variation in solidarity along age and partisanship lines. Another set of studies examines preferences towards vaccination prioritization within countries and across population subgroups (Ceccato et al., 2021; Duch et al., 2021; Gollust et al., 2020; Knotz et al., 2021a; Luyten et al., 2022; Persad et al., 2021; Reeskens et al., 2021; Schaeffer and Haderup Larsen, 2022). Our work contributes to this second stream.

Previous studies have largely leveraged ranking tasks and forced-choice setups to assess the role of individual and group characteristics in COVID-19 vaccine allocation preferences. Most studies have employed age, occupations, and health cues of vaccine candidates as cross-cutting features (see Table 2 for an overview). Much existing evidence, therefore, speaks to the question of whether the public adheres to popular principles and practices. There are, however, further attributes of inquiry specific to single studies, such as ethnic cues (Schaeffer and Haderup Larsen, 2022), healthy lifestyles, and compliance with COVID-19 containment guidelines (Reeskens et al., 2021). Those studies provide additional insight into whether public prioritization preferences are also shaped by non-utilitarian considerations.

**Table 2:**
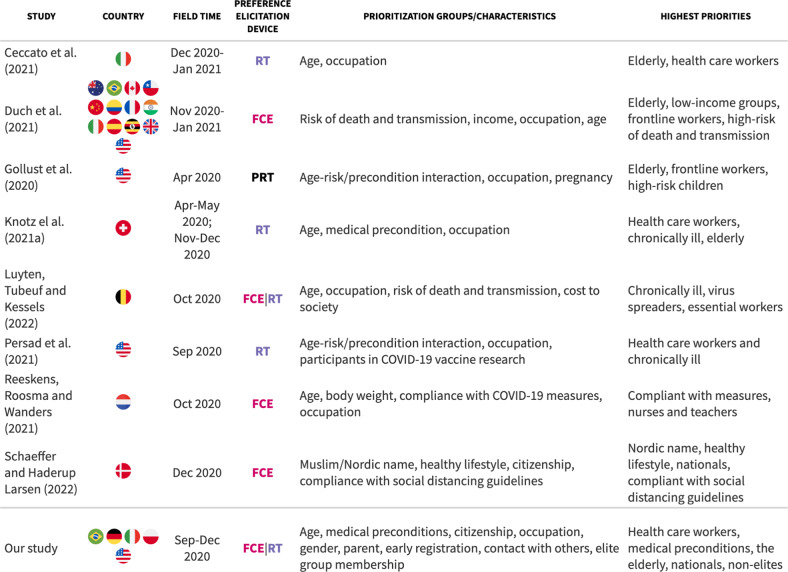
Overview of studies on COVID-19 vaccination allocation preferences.

*FCE* forced-choice experiment, *RT* ranking task, *PRT* priority rating task.

In an early timed survey that was fielded in the United States in April 2020, Gollust et al. (2020) present results from a priority rating task. The respondents had to categorize each of eight groups (frontline workers, children with serious illness, people aged 65 years and older, middle-aged people with serious illness, essential workers who interact with the public, pregnant people, children with moderate risk, adults with moderate risk) as low, medium or high vaccination priority. The study reports highest prioritization levels for frontline staff as well as high-risk children and the elderly.

In another early survey fielded in Switzerland between April and May 2020 (and again between November and December 2020), Knotz et al. (2021a) used a ranking task of several target groups (children, elderly, chronically ill, health-care workers, police officers, teachers, and the general population). The authors find public support for prioritizing health-care workers and the elderly, but also evidence for inter-generational solidarity (young cohorts prioritizing the elderly and vice versa).

Persad et al. (2021) implemented a ranking task of priorities in two surveys fielded in September 2020 in the US. They find broad consensus between public preferences and the recommendations of the Advisory Committee on Immunization Practices (ACIP) and the National Academies for Science, Engineering, and Medicine (NASEM); health-care workers and adults with serious comorbid conditions are ranked highest but so are communities that have been disproportionately affected by the pandemic (Black, Hispanic, Native American). However, the public was less in favor of prioritizing healthy older adults, an effect driven by older respondents and somewhat at odds with previous findings (Gollust et al., 2020).

Reeskens et al. (2021) implemented a forced-choice conjoint experiment that was fielded in an online household panel in the Netherlands in October 2020. Profile characteristics included age, obesity, compliance with COVID-19 measures, and occupation. The authors find that the Dutch sample prioritized those employed in crucial sectors, but also heavily penalized those disobeying public health measures. Interestingly, there was no difference between the young and the old profiles.

Luyten et al. (2022) report results from a ranking task and a conjoint experiment in Belgium in October 2020. This study elicited ranking preferences over eight prioritization strategies, including prioritizing chronically ill, the elderly, essential professions, but also a lottery and the first-come, first-served principle, as opposed to demographic groups. This revealed three dominant strategies—to prioritize the chronically sick, essential professions, and the elderly.

In the most comprehensive study of public COVID-19 vaccination allocation preferences to date, Duch et al. (2021) report results from a conjoint experiment implemented in 13 countries between November 2020 and January 2021, letting participants evaluate potential vaccine recipient profiles with the randomly varying attributes of occupation, age, risk of contracting and transmitting the virus, risk of death from COVID-19, and income. They find the public to prioritize the elderly, low-income groups, key workers (health care, education, police), and those with high transmission risk, and high vulnerability. Despite substantial variation in allocation policies across countries in the sample, they find preferences to be largely consistent across populations.

Schaeffer and Haderup Larsen (2022) implemented a paired vignette with a forced-choice experiment in an online survey in Denmark during the first week of the vaccination program (December 2020). Ethnic minorities (patients with Muslim names) and those who recently immigrated were seen as lower priority for vaccination. Patients portrayed as not following a healthy lifestyle or social distancing guidelines were also penalized.

Overall, existing evidence suggests that in most contexts respondents tend to prioritize the elderly, frontline health-care workers, and individuals with medical preconditions for access. However, there is also evidence for non-utilitarian principles at work. Perceived deservingness has been found to be influenced by compliance behavior and lifestyle (Reeskens et al., 2021; Schaeffer and Haderup Larsen, 2022), as well as vaccination chauvinism (Schaeffer and Haderup Larsen, 2022). Our study adds to the existing literature in three ways: First, we provide additional cross-national evidence, holding the design constant across various contexts. Second, we pursue a variety of methodological approaches, which allows us to explore in a more nuanced fashion the role of different group and individual characteristics that would not be implementable in a ranking or choice task alone. Third, we introduce characteristics that have only rarely (or not at all) been studied before, including elite group membership, citizenship, parenthood, and the first-come, first-served principle. In the following section, we motivate our approach and expectations further.

## Data and methods

### Case selection and subject recruitment

We selected five countries to field our survey: Brazil, Germany, Italy, Poland, and the US. The study was carried out prior to the roll out of vaccines in all five countries. The cases were selected to exhibit variations in the trajectory of COVID-19 infection rates, and the government’s ability to provide intensive care for critically ill patients. At the time of data collection, the five countries in our sample were experiencing very different infection and death rates. Brazil and the US had some of the highest levels of infection and COVID-related deaths worldwide, whereas the European countries still had only low levels. Poland had experienced very few cases up to this point, but Germany and particularly Italy experienced a first wave of increased infections in April 2020. We chose countries that also varied significantly in their capacity to deal with serious COVID-19 infections using the number of beds in intensive care units as a proxy.^3^

Lastly, we chose to include countries that presented at least some variation in their initial national vaccination priority policies (see Table 1). Germany and Italy were the only countries to initially include individuals with high-risk contacts in their priority list, Brazil was the only country to include marginalized groups, and the US was the only country to include non-healthcare-related frontline workers. Poland had the fewest number of priority groups.

The survey sample includes 4366 individuals from the five countries from September to December 2020, recruited from a commercial access panel run by Respondi AG and using quotas to approximate marginal distributions of age, gender, and education in the adult populations of each country. The study is part of a two-wave survey (three waves in Germany) that was originally launched in August 2020. The instruments on vaccine distribution preferences were included in the second wave (third wave in Germany). The sample size varies between countries (BR = 799, DE = 1651, IT = 1002, PL = 803, US = 498). The survey design and programming were implemented by the authors.

### Design and measures

To elicit vaccine prioritization preferences, we rely on two tasks: a priority ranking question of pre-defined societal groups and a conjoint experiment with pairs of hypothetical subjects, one of whom must be prioritized for vaccination. The two tasks complement each other. The ranking task corresponds directly to the public discourse and official guidelines, which also ranked people based on group membership. The task also matches the prioritization logic ("sooner” vs. “later”) and lets respondents compare multiple groups efficiently. However, the ranking task does not isolate the effect of group attributes (like age, profession, health status, etc.). The conjoint experiment can isolate the attribute effects and measure priorities over the kinds of individual characteristics that might be considered in a concrete trade-off. Conjoint experiments are a common approach to understanding how people value different characteristics and are increasingly used to capture policy preferences in the public (Gallego and Marx, 2017; Horiuchi et al., 2018; Ryan and Farrar, 2000). They are also useful for mitigating social desirability bias, as multiple attributes are manipulated simultaneously (Horiuchi et al., 2021). Consistency of results across different tasks strengthens our findings.

#### Ranking task

**Fig. 1:**
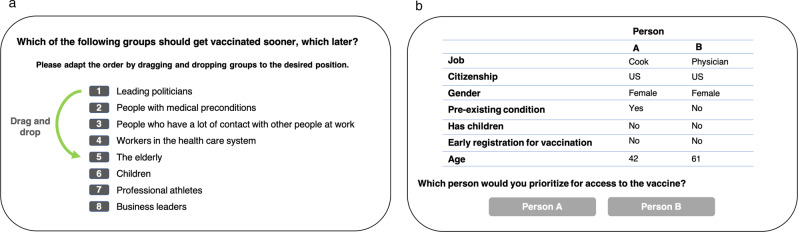
Illustration of ranking task and conjoint experiment. **a**: Rankings of preferred priorities. **b** Example setup of forced choice conjoint experiment.

For the ranking task, respondents ranked a set of eight groups against each other. Panel (a) in Fig. 1 illustrates the task. Groups varied according to age (children, elderly), profession (health-care system workers, politicians, athletes, business leaders, people who have a lot of contact with others at work), and health (medical preconditions). Respondents were asked to position the groups in descending order, starting with the group that should get vaccinated soonest. The initial order of the groups was randomized across subjects.

As the experiment was fielded before vaccines became publicly available, we relied on proposals from experts to construct groups for the ranking task (see Dooling et al., 2020; Gemeinsame Arbeitsgruppe der Mitglieder der Ständigen Impfkommission, 2020). In these proposals, the principles underlying prioritization were (a) to minimize severe or fatal COVID-19 cases, (b) to protect workers in jobs with a high risk of exposure, (c) to avoid transmission within vulnerable populations, and (d) to maintain public life and ensure government effectiveness. Based on these principles, the experts recommended prioritizing persons of higher age and with medical preconditions, workers in the health-care sector, and workers who have a key position in basic areas of public service and for maintaining central state functions. To these categories, we added leading politicians, professional athletes, and business leaders to allow us to contrast groups already prioritized with others that could be seen as worthy under a different utility rationale; considering leading politicians as a group of people who have a key position in maintaining central state functions, and professional athletes for public entertainment. We added children, as they are commonly regarded as particularly worthy of protection even though they are not considered a high-risk group.

#### Conjoint experiment

The conjoint experiment presents pairs of hypothetical profiles to each study participant, who was asked which individual in each pair should be prioritized for vaccination (see Panel (b) in Fig. 1). The hypothetical profiles were constructed as combinations of the following characteristics: gender (female, male), age (27, 42, 61, or 76 years), children (has children, does not have children), occupation (nurse, physician, teacher [only in the German survey], cook, professor, unemployed), citizenship (citizen, non-citizen), pre-existing medical condition (yes, no), and early registration for vaccination (yes, no).

As with the ranking task, we combined a set of attributes that reflected characteristics recommended by experts (age, pre-existing condition, profession) with others that implied heuristics that were not endorsed by most experts. The citizenship attribute was added to probe nativist responses. The gender and children attributes allow us to identify discrimination against females vs. males and parents vs. non-parents, respectively. Finally, we added the attribute “early registration for vaccination” to explore whether respondents would prefer a “first-come, first-served” strategy, as this was considered in other ethical dilemmas during the pandemic (Herreros et al., 2020).

Each participant evaluated four pairs of hypothetical individuals with varying attributes. The attribute levels were completely randomized. The order of the attributes was randomized between participants so they would see attributes in the same ordering for all profiles. After completing the task, participants were offered an open field to reflect on the exercise and state what characteristics of a person they considered to be the most important for vaccine prioritization. This provides us with an additional measure of the relevance of different attributes in participants’ decision-making.

#### Covariates

We use a set of individual-level covariates to perform subgroup analyses and extract average marginal interaction component effects (AMICE). *age* is measured in five categories: (18–29 years, 30–39 years, 40–49 years, 50–59 years, 60+ years), *gender* (male = 0, female = 1) and *children* (yes/no) are binary responses. We divide an 11-point left-right ideological scale into three categories (left = [1–4], moderate = [5–7]), and right = [8–11]) to create a *political lean* variable. Finally, we consider *pre-existing health conditions* based on whether respondents “have any pre-existing conditions that increase the risk of a severe course of COVID-19 (e.g., high blood pressure, obesity, diabetes, COPD)” and encoding their answers to yes/no.

### Analyses

We use three approaches to evaluate respondents’ preferences for vaccine priority access.

In the group ranking task, we estimate a group’s average priority ranking. The resulting scores are a simple average based on the mean position of the group and an absolute rank (1–8).

To study the role of individual profile attributes in the conjoint experiment, we estimate marginal means, average marginal component effects (AMCE), and average marginal interaction component effects (AMICE) using linear regression models with clustered standard errors.

Finally, to identify the importance of individual profile attributes, we examine (a) the contribution of each attribute to model fit when variables are included in a random forest and (b) responses to an open-ended survey question about the relevance of different attributes in participants’ decision-making. We present all results by country and pooled (weighted for sample sizes).

## Results

### Ranking groups by priority

**Fig. 2:**
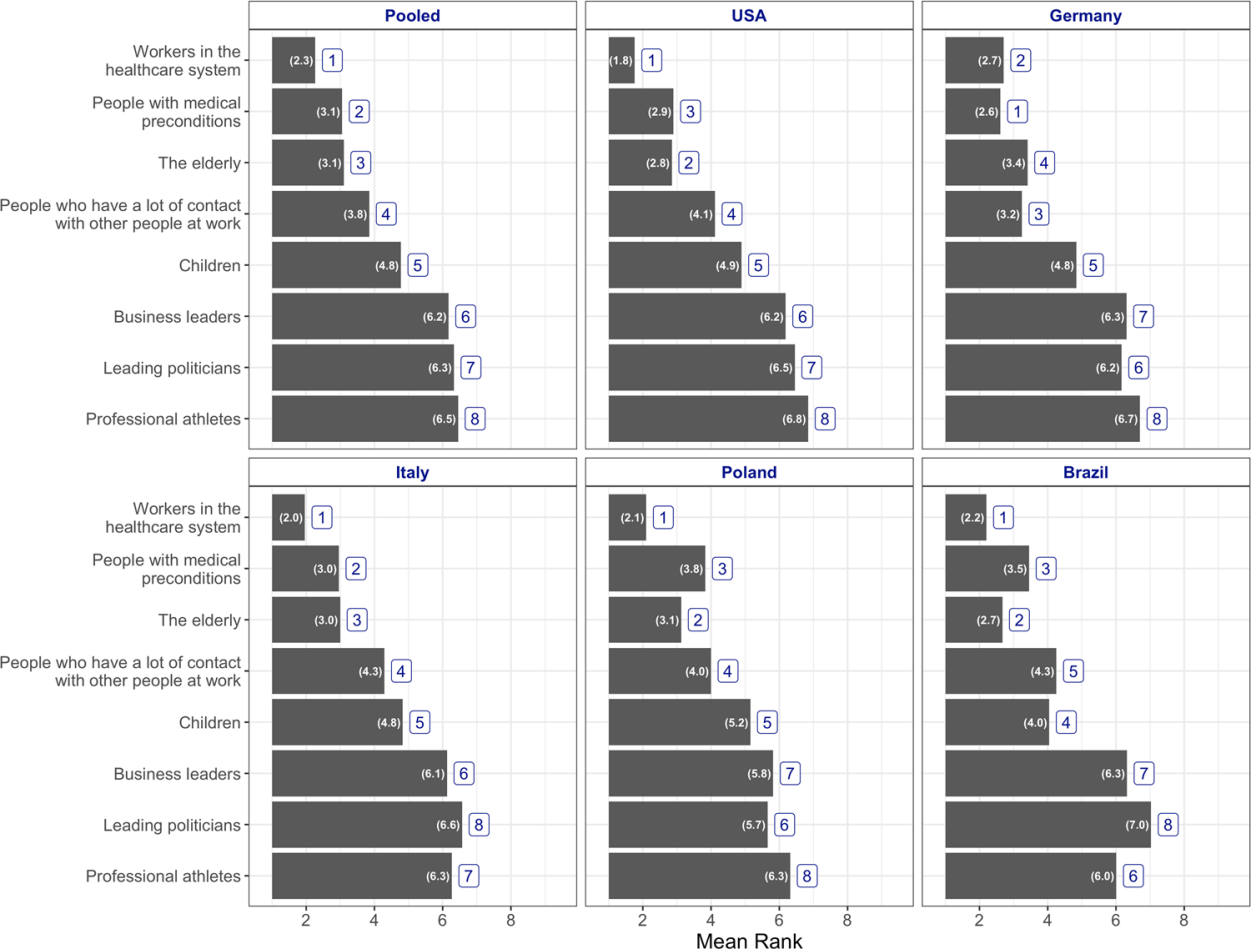
Average ranks of groups by preferred priority. (“Which of the following groups should get vaccinated sooner, which later?”). Note: numbers inside the bar represent mean rank. Numbers outside the bar represent the absolute rank for each group.

The results from the rank-order task present a similar picture across the different countries. Figure 2 shows that respondents prioritized workers in the health-care system. The group has an average ranking of 2.3, the top-ranked group in absolute terms across national samples. Additionally, in all countries but Germany, workers in the health-care system, people with medical preconditions, and the elderly were the top three highest priority groups.

The pooled measure suggests a modest cluster in the bottom average rankings around the sixth mean rank between professional athletes, leading politicians, and business leaders. While these three groups consistently rank lower, the politician category is the modal choice for the eighth ranking. Supplementary Figs. 15 and 16 illustrate the distribution of the ranking choices. Children, a part of the population largely absent from vaccination priority ordinances, are consistently positioned in the middle of the ranking, occupying fourth and fifth place in most countries.

### The relative value of individual characteristics

**Fig. 3:**
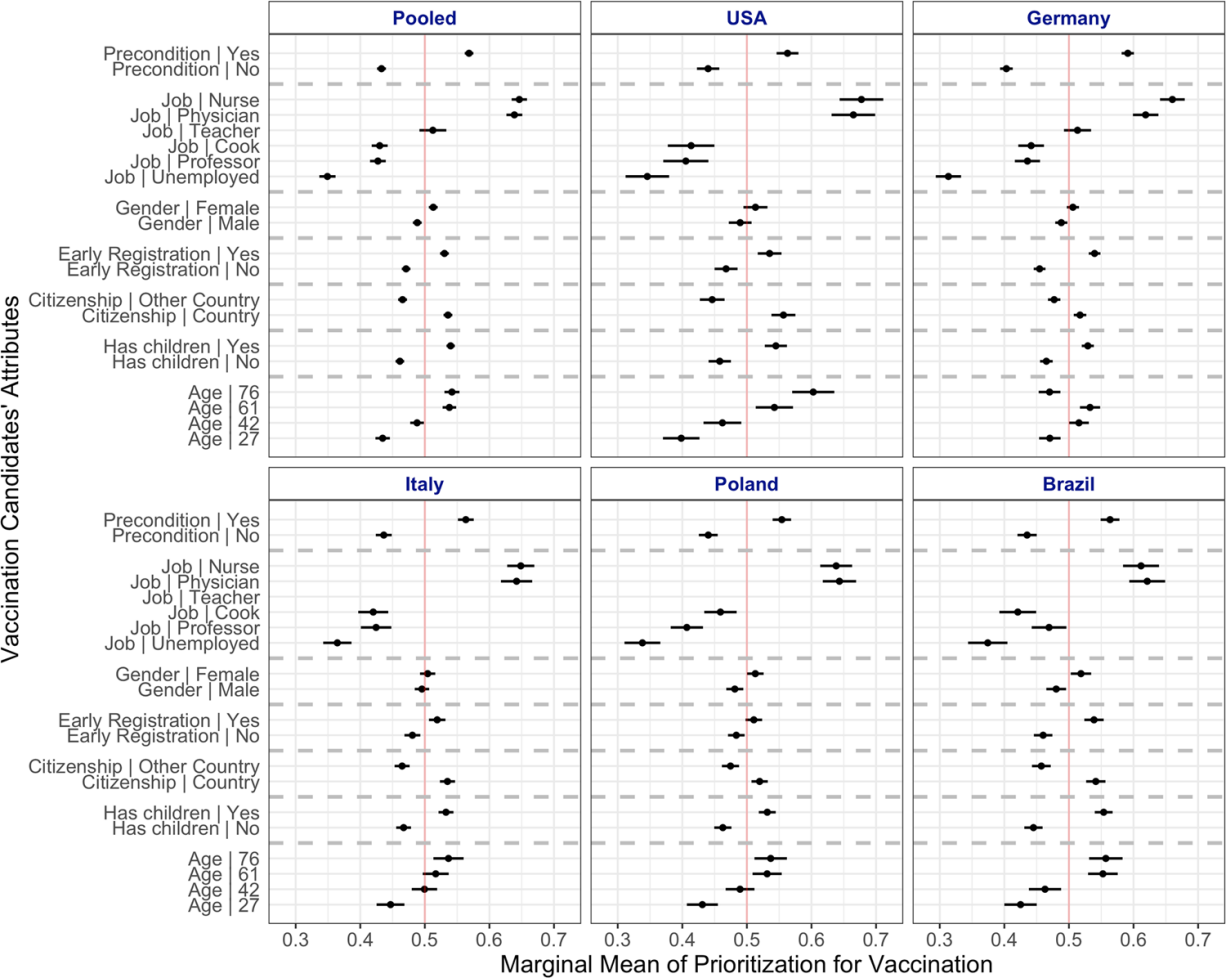
Effects of vaccination candidates' attributes on the probability to be prioritized in conjoint task. Marginal means with 95% confidence intervals displayed for the five countries and pooled samples.

The findings from the conjoint experiment reinforce the picture of homogeneity of prioritization preferences across the national contexts of our study. Figure 3 presents the marginal means for the different vaccination candidate attributes in the five countries, and the sample size-weighted pooled effects. These results are analogous to the average marginal component effects (AMCE) illustrating the estimated change in selection probabilities of the characteristics in the respondents’ choice against an attribute baseline presented in Supplementary Fig. 8.

As in the rank-order task, workers in the health-care sector and those with preconditions are prioritized. Regardless of other characteristics, nursing staff and physicians had about a 30 percentage points (pp) higher probability of being selected than unemployed candidates, and still between 10 and 20pp higher probability than cooks and professors. A candidate with medical preconditions had an about 15pp higher probability of being selected over a similar candidate without preconditions.

We observed less pronounced differences in favor of candidates with children, citizens of the country, early registration, older candidates, and women. In most countries, candidates falling between the two above-60 groups (ages 61 and 76) had on average about 10pp higher probability of being chosen compared to a corresponding 27-year-old candidate across samples. The German sample is the only exception; candidates in the middle categories (ages 42 and 61) were more likely to be prioritized than those in the 27 and 76-year-old categories.

### Attribute importance

To gather further insight from the conjoint experiment, we examine two measures of attribute importance. In addition to extracting the change in selection probabilities against a trait-specific baseline with the marginal means, we can also explore which attributes carry more weight in the participants’ decisions. First, we report on the results of the attribute importance when using random forests to model the decision-making process in the conjoint experiment. The approach gives us an indication of how predictive different attributes are for respondents’ decisions. In contrast to the marginal effects presented above, variable importance is intended as a measure of how useful knowing each profile attribute is to individual decisions as a whole rather than how different levels of the same attribute affect decisions.

The random forest is an ensemble of decision trees, for which we can analyze the contribution of different attributes to the model fit when the attributes are included in the forest. We use the mean reduction in the GINI as a measure of variable importance. The GINI measures the purity gain by splitting at a given attribute. When variables are useful, it tends to correctly classify patients to determine if they are prioritized. When attributes are not useful and do not contribute much to a correct classification of the prioritization decision, they are less important in explaining the decisions. The evaluation of attribute importance via random forests confirms the general inclinations from the rank-order task and the changes in probabilities derived from the conjoint experiment (see Fig. 7 in Supplementary Appendix). The attributes that dominate the measure are, once again, professional, specifically of the medical sector (nurses and physicians), and health related (preconditions).

Next, we examine the text of the answers to the open question: “Please think again of the questions you just answered. What characteristics of a person would be most important to you in deciding who should have access to a vaccine first? Note: You can name characteristics mentioned in the questions just answered as well as other characteristics.” To perform the text analysis, we rely on a dictionary containing words and phrases signaling membership to a category (for a detailed description see Supplementary Appendix 3.1). Around 40% of respondents signaled in their open question response that old age was the most important characteristic in deciding who should get vaccinated first (Supplementary Table 2). This stands in contrast to the measure of attribute importance using random forests, which suggests the contribution of the candidate’s age in decisions was only modest. This discrepancy may indicate that respondents are not always aware of the heuristics they apply when making decisions.

### Preference heterogeneity

The public could still be highly divided within countries even if priorities appear to be similar across national contexts on average. Considerable within-country disagreement would undermine the portrayal of shared cross-national prioritization preferences. However, within-country heterogeneity cannot be inferred from the results presented so far, as the estimates average over respondents with potentially different response patterns, which might even cancel each other out. To explore this possibility, we compute the average marginal interaction conditional effects (AMICE) and subgroup analyses for a set of demographic- and ideology-based covariates. For the demographic characteristics, we investigate whether respondents prioritized profiles that shared their attributes (sex, parenting, and health status). For example, do parents in the panel prioritize candidates with children? Or, do respondents with medical preconditions prioritize those in a similar situation? For ideology, we explore respondents’ political ideology (left, moderate, and right), as well as a scaled measure of institutional trust (low, medium, and high).

In our exploration of preference heterogeneity, we do not find evidence that effects are conditional on these respondent characteristics (see Supplementary Figs. 9–14). We only find modest heterogeneity based on respondents’ political leaning and the citizenship status of the candidate. A candidate who is a citizen of the country has about 10% higher probability of being chosen over a similar non-citizen by right-leaning participants, compared to about 3% by left-leaning respondents. These differences are more salient in the Italian, US-American, and German contexts. Nevertheless, citizenship status has low attribute importance according to the measures reported above, so the conditional effect is less important when judging the coherence of the public’s priorities.

Overall, we find no clear indication of heterogeneous effects with respect to participants’ own attributes and conclude that public prioritization preferences are reasonably coherent within countries. In short, we see no strong polarization over COVID vaccination priority.

## Discussion and conclusion

Vaccination in the context of the COVID-19 pandemic is inevitably a question of life and death, and created a situation in which individuals are in competition for their fundamental right to health. In situations of vaccine scarcity, one person receiving the vaccine means another goes without, at least for some time. Given the gravity of these consequences, the decision about whose vaccination to prioritize is not easily delegated to the public—nor should it be. Nevertheless, the success of such vaccination schemes ultimately relies on high vaccination rates which in turn depend on public perception about how fairly vaccines are allocated (see Duch et al., 2021, p. 2), making it particularly important to understand them.

Our cross-national study shows that respondents’ preferences largely reflect the guidelines of medical associations issued just before the first COVID-19 vaccines became available. Regardless of where they live and what other individual preferences they have, respondents clearly prioritize frontline workers in the medical sector (nurses, physicians), people with medical preconditions, and the elderly. This finding suggests that the public prefers those who are most likely to be severely impacted by an infection, and those who are most needed to mitigate the most adverse effects of the pandemic on the country. This is largely line with expectations based on utilitarian principles as well as with recent evidence from other studies (Duch et al., 2021; Gollust et al., 2020; Knotz et al., 2021a; Luyten et al., 2022; Persad et al., 2020; Reeskens et al., 2021).

Importantly, there are other factors that matter but which are not considered in public guidelines: First, respondents prioritize individuals who signed up early for a vaccine, reflecting a “first-come, first-served” heuristic. Second, parents are prioritized over non-parents. Third, we observe a penalty effect for non-citizens in all settings, suggesting health-care chauvinism among the respondents. This is consistent with studies on discrimination against immigrants, non-citizens, but also political opponents in a similar setting of access to life-saving health care during the pandemic (Helbling et al., 2022; Knotz et al., 2021b; Larsen and Schaeffer, 2021; Stoetzer et al., 2022). This finding, together with respondents’ revealed preferences for public elites not to be prioritized, highlights that respondents’ perceptions of deservingness diverge in important ways from implemented practice in vaccination prioritization.

It is important to point to some limitations of our study. First, while we do offer comparative evidence and find that empirical patterns are very similar across the countries and subgroups, they do not necessarily travel to other contexts. Nevertheless, our findings are consistent with Duch et al. (2021), who find a broad consensus on which population attributes should have priority in a sample of 13 countries, and a comparative assessment of the results of multiple single-country studies seems to support this finding. Second, our sample is restricted to individuals who opted into an online access panel. While their marginal distribution of demographics roughly matches our populations of interest, they might differ on unmeasured attributes that affect preferences, such as knowledge about public prioritization guidelines. Third, our preference measures might be biased by social desirability. This could happen when respondents regard public guidelines as the gold standard of what is socially acceptable and state preferences accordingly. Our conjoint design may help mitigate this social desirability bias (Horiuchi et al., 2021). Indeed, we do find evidence for preferences that could be considered as socially undesirable (health-care chauvinism penalizing non-citizens). Finally, our results are not informative about actual prioritization by those who have the power to decide in practice (the medical personnel who perform the vaccinations), so we cannot say anything about actual discrimination in vaccination allocation.

With these limitations in mind, our findings show that public preferences are largely in line with ethical guidelines that had been issued early in the process of creating allocation policies for COVID-19 vaccines. While many decisions and approaches to contain the pandemic have been met by protest and opposition, the broad consensus about the key criteria for vaccination prioritization highlights an opportunity for policymakers to point out that this particular action is consistent with the will of the public.

Our findings also reveal that preferences of ordinary citizens can include discrimination against subgroups based on non-utilitarian grounds. Neither the prioritization of citizens over non-citizens nor following the first-come, first serve principle are in line with public guidelines. While it is important that policymakers become aware of these differences to better be able to increase acceptance of vaccination programs, it is also clear that responsiveness has its limits when fundamental human rights are at odds with public preferences.

Beyond the COVID-19 pandemic, our study contributes to research on public preferences on policy and regulatory action more broadly. Policy decisions often involve the allocation of scarce resources. Understanding the extent to which these decisions overlap with public opinion (while not disregarding fundamental rights concerns) can help policymakers design responsive programs while strengthening the legitimacy of their work. Against this backdrop, the study of COVID-19 vaccination allocation preferences is an encouraging example of interdisciplinary research in the social and behavioral sciences to inform policies (Bavel et al., 2020) where evidence across multiple contexts has accumulated quickly.

## Supplementary information

Supplementary Information

## Data Availability

The data and computer code for this study are open source and available on this Dataverse repository: 10.7910/DVN/OAMAOE

## Footnotes

1 It took less than a year from the start of the pandemic to the start of production and distribution of the world’s first vaccines Polack et al. (2020), Thanh Le et al. (2020). According to the World Health Organization (WHO), by July 2022, there were 366 vaccine projects, of which 168 were at the clinical development stage (World Health Organization, 2022).

2 Notably the Strategic Advisory Group of Experts on Immunization (SAGE) at the WHO created a set of guiding normative values, vaccine-specific recommendations, and a vaccine prioritization road-map to support countries in the development of their national plans World Health Organization (2021).

3 While these data are not available for all countries, an OECD comparison shows that Germany and the US are at the top of the list, whereas Italy and Poland are towards the bottom of the list. Brazil, as a non-OECD country, has a capacity that is comparable to Italy and Poland Palamim and Marson (2020).
