# Supplementary material for: Prioritization preferences for COVID-19 vaccination are consistent across five countries
Source: Humanit Soc Sci Commun. 2022 Dec 7;9(1):439. doi: 10.1057/s41599-022-01392-1 (PMC9735138; doi:10.1057/s41599-022-01392-1)

Prioritization preferences for COVID-19 vaccination are consistent across five countries

Supplementary Information

1 Research Design

1.1 Ethical Considerations

The study was approved by the Dean's office at the Hertie School which serves in lieu of an ethics committee. Our research complies with GDPR requirements and all relevant ethical regulations, as documented in the German Research Foundation's Code of Conduct Guidelines for Safeguarding Good Research Practice. The research also adheres to the Principles and Guidance for Human Subjects Research approved by the APSA Council. In the following we discuss some additional considerations:

Participant recruitment and compensation: In partnership with the online survey firm Respondi AG, panelists were invited to participate in a study on attitudes in the context of the COVID-19 pandemic. Participation was voluntary. The survey design and programming were implemented on our end. The access panel provider and its local partners compensate their participants with their standard rates of cash transfers or platform-specific currencies for voluntary participation. In our case, participants were compensated with 0.50€ in the US, 0.75€ in Poland and Germany, and 17 Korpus points in Brazil and Italy, for their participation in the 15 minute survey.

Informed Consent: At the beginning of the survey, we obtained voluntary and informed consent from participants. We explain the research project, study purpose, the risk and benefits and described direct contact details to inquire further information. Supplemental Figure 1 and 2 provides a screenshot of the text as presented to the (US) participants. Participants in other countries received a translated but otherwise identical version.

Confidentiality: The identities of research participants are kept confidential. Contacting details are kept with the survey firm Respondi. The anonymized response data is stored with the researchers. The anonymized data will be shared with the research community.

Deception: The research design does not use any form of deception.

1.2 Survey Questions

This section provides the English wording for the survey questions we employed in our analysis. The questionnaires were translated by a native speaker in their respective language.

- **Age:** What is your year of birth? [*Scroll menu*]
- **Gender:** Please state your gender [*Male*, *Female*, *Other*]
- **Children:** Do you have children? [*No*, *Yes, 1 child*, *Yes, 2 children*, *Yes, 3 children*, *Yes, 4 or more children*]
- **Political lean:** In politics people often talk about the "left" and the "right". On a scale between 1 (furthest left) and 11 (furthest right), where would you place yourself? [*Scale 1-11*]
- **Pre-existing health conditions:** Do you have any pre-existing conditions that increase the risk of a severe course of COVID-19 (e.g., high blood pressure, obesity, diabetes, COPD? [*Yes*, *No*, *Don't know*]
- **Trust:** How much trust do you have in the following groups and institutions? [Items: (State government, media, federal government, scientific experts, and the healthcare system) [*Scale 1-5 (Not trust at all-Complete trust*]

2 Survey screenshots

Supplementary Figure 1: Screenshot of welcome screen

Welcome back!

Today, we would like to invite you to participate in a study conducted by researchers from the [redacted]

You have already participated in the previous survey - thank you! Today, we would like to learn more about your personal situation as well as your opinions in the context of the COVID-19 pandemic.

Your participation is voluntary. You are free to not answer any question or to withdraw from the study at any time. This poll should take approximately 10 minutes to complete.

We hope that you would like to take part again!

Supplementary Figure 2: Screenshot of informed consent

Consent

Before you decide to participate in this study, it is important that you understand why the research is being done and what it will involve. Please take the time to read the following information carefully. Please contact the researchers if you need more information.

Research Project

The title of the research project is "Life in Times of COVID-19". This research project is being conducted by [redacted]

Purpose of the Research

This is a study on public opinion and behavior during the COVID-19 pandemic. Citizens in multiple countries are surveyed. Your participation is voluntary. You have been invited to and participated in a previous survey on the same topic. Participation involves completion of a follow-up survey. You may choose not to answer any or all questions.

Study Procedure

You will be asked a series of questions in an online survey - mostly you will answer by clicking boxes to supply your opinion or experience. You may choose not to answer any questions that you are not comfortable answering, and you can withdraw from the survey at any time. Your answers to this survey will be merged and analyzed together with the answers of the previous survey you participated in.

Risks and Benefits

Risks to participation are minimal, and while there are no direct benefits, you will be helping to further scientific understanding of current public opinion and behavior. Researchers involved in the study will protect your personal information, and others will not be able to connect your responses to personally identifiable information. Any personal information that could identify you will be removed or changed before files are shared with other researchers or results are made public.

Contact

Questions about this project may be directed to [redacted] Any concerns can be directed to [redacted]

I hereby confirm that I am at least 18 years old, that I have read, understood, and agree with the terms detailed above. Do you wish to participate in this survey?

Yes

No

Supplementary Figure 3: Example of ranking task

As soon as a vaccine against COVID-19 becomes available, it is likely that everybody cannot be vaccinated at once.

What do you think, which of the following groups should get vaccinated sooner, which later? Please put the group you think should be vaccinated first to the top and the group you think should be vaccinated later to the bottom.

Please adapt the order by dragging and dropping groups to the desired position.

Business leaders

2 Leading politicians

Professional athletes

People with medical preconditions

Children

The elderly

Workers in the health care system

People who have a lot of contact with other people at work

Supplementary Figure 4: Screenshot of conjoint decision description

Please read the following very carefully.

Once a vaccine against COVID-19 becomes available, it is likely that the initial demand is higher than what the vaccine producers can supply. Individual characteristics may play a role in who gets access to the vaccine first. How would you determine who gets priority in receiving the vaccine?

In the following you will see pairs of persons with differing characteristics. Please look carefully at the two profiles and tell us which person you would prioritize for access to an effective and safe COVID-19 vaccine.

Supplementary Figure 5: Example of conjoint decision

Please look at the profiles of the two persons thoroughly and then make your decision.

Note: Please assume that the vaccine is both safe and effective, i.e. does not have serious adverse effects and protects the person reliably from getting COVID-19.

	**Person**	
	**A**	**B**
**Has children**	Yes	Yes
**Gender**	Male	Male
**Age**	76	27
**Pre-existing condition**	No	No
**Early registration for vaccination**	Yes	No
**Job**	Nurse	Unemployed
**Citizenship**	US	US

Which person would you prioritize for access to the vaccine?

Person A

Person B

Supplementary Figure 6: Example follow-up open-ended question

Please think again of the questions you just answered.

What characteristics of a person would be most important to you in deciding who should have access to a vaccine first?
Note: You can name characteristics mentioned in the questions just answered as well as other characteristics.

3 Supplementary Tables and Figures

3.1 Attribute importance

Supplementary Figure 7: Evaluating attribute importance using random forests

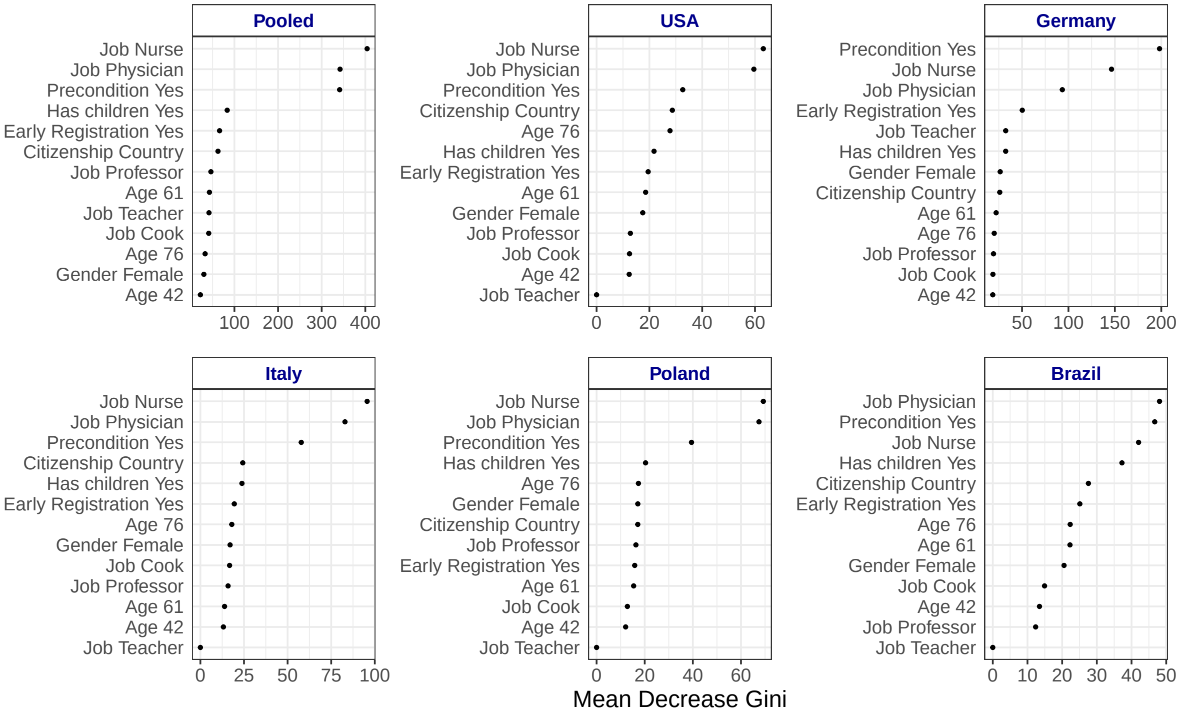

Attribute Importance from Open Question Answers: To analyze the content of the open question, we employed a custom dictionary outlines in Supplementary Table 1. We developed the based on the original English-language U.S. responses and machine translations of the responses of the other countries. We employed the complete vocabulary list of each country's responses. Supplementary Table 2 presents the distribution of responses at the country- and pooled-level.

Supplementary Table 1: Custom dictionary for open question attribute importance

Attribute	Keywords and Phrases
Old age	elderly, age, over_65, senior_citizen, elder, elders, old, over_70
Life expectancy	young, life_expectancy
Occupation	occupation, job, doctor, doctors, work, workers, profession, nurse, physician, frontline, career, teacher, teachers, essential, world_leaders, system_relevant, police
Preconditions	preconditions, precondition, health, conditions, history, immune, disabled, illness, pre-illness, pre-existing, ill, at-risk, comorbidities, comorbid, chronic
Has children	has_children, children_to_raise, had_kids, have_children, with_children, with_kids, has_younger_children, has_small_children, family, dependent, parent, dependents, parents, mother, father, family_man, having_children, age_of_children
Registration	registration, registered, sign_up, first_come, enrolled
Exposure	exposure, contact, contacts
Productivity	merit, contribution, education, service, value_in_society, benefit_to_the_general_public, productivity, function_for_society, knowledge, who_contribute, expected_gains
Citizenship	citizen, us_born, american, americans, citizenship, nationality, country, german, germans, brazilian, brazilians, polish, pole, italian, italians
Income	income, purchasing_power
Express interest	want_it, want_the_vaccine, everyone_who_wants
Gender	male, female, gender

Supplementary Table 2: Open question response breakdown. Mentions are shown in counts and as a percentage of respondents.

	Pooled	BRA	DEU	ITA	POL	USA
Old Age	1726 (39.5%)	263 (40.7%)	610 (38.5%)	354 (37.1%)	306 (41.2%)	193 (44.3%)
Preconditions	1537 (35.2%)	243 (37.6%)	592 (37.4%)	280 (29.3%)	236 (31.8%)	186 (42.7%)
Occupation	1164 (26.7%)	138 (21.4%)	417 (26.3%)	267 (28%)	197 (26.5%)	145 (33.3%)
Exposure	221 (5.1%)	21 (3.3%)	90 (5.7%)	58 (6.1%)	37 (5%)	15 (3.4%)
Has Children	201 (4.6%)	42 (6.5%)	47 (3%)	37 (3.9%)	46 (6.2%)	29 (6.7%)
Registration	110 (2.5%)	23 (3.6%)	70 (4.4%)	2 (0.2%)	5 (0.7%)	10 (2.3%)
Citizenship	91 (2.1%)	10 (1.5%)	31 (2%)	13 (1.4%)	15 (2%)	22 (5%)
Productivity	50 (1.1%)	4 (0.6%)	14 (0.9%)	6 (0.6%)	20 (2.7%)	6 (1.4%)
Life Expectancy	32 (0.7%)	5 (0.8%)	7 (0.4%)	13 (1.4%)	7 (0.9%)	0 (0%)
Gender	9 (0.2%)	3 (0.5%)	2 (0.1%)	0 (0%)	1 (0.1%)	3 (0.7%)
Express Interest	5 (0.1%)	0 (0%)	1 (0.1%)	0 (0%)	0 (0%)	4 (0.9%)
Income	4 (0.1%)	2 (0.3%)	0 (0%)	0 (0%)	0 (0%)	2 (0.5%)
No response	379	92	128	74	72	13
Total	4365	646	1585	955	743	436

3.2 Average Marginal Component Effects (AMCE)

Supplementary Figure 8: Change in selection probabilities by individual characteristics (compared to baseline characteristic).

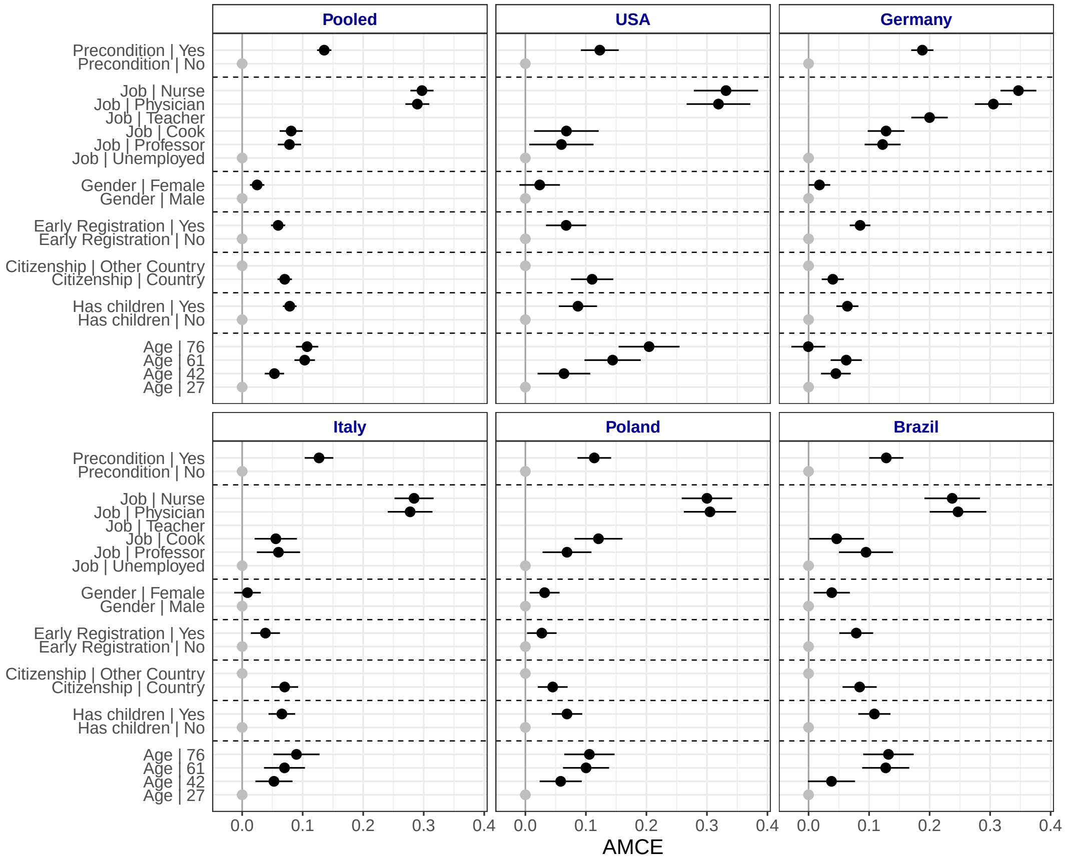

Note: Average marginal component effects and 95% confidence intervals for prioritizing individuals in the five country and combined samples.

3.3 Subgroup Analyses and Average Component Marginal Interaction Effects (ACMIE)

Supplementary Figure 9: Subgroup analysis. Change in selection probabilities by individual characteristics conditional on respondent's scaled measure of institutional trust

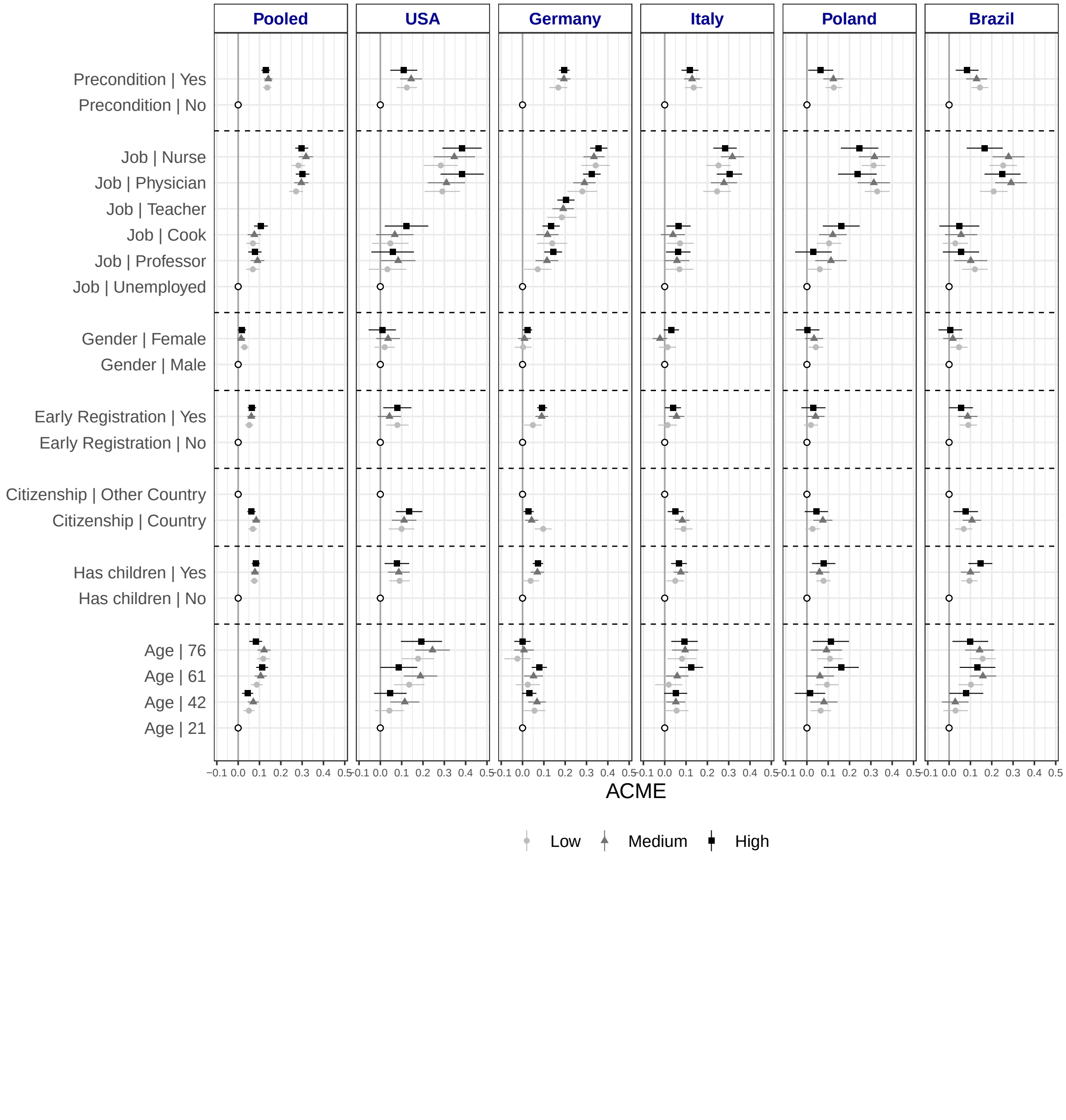

Supplementary Figure 10: Subgroup analysis. Change in selection probabilities by individual characteristics conditional on respondent's political ideology

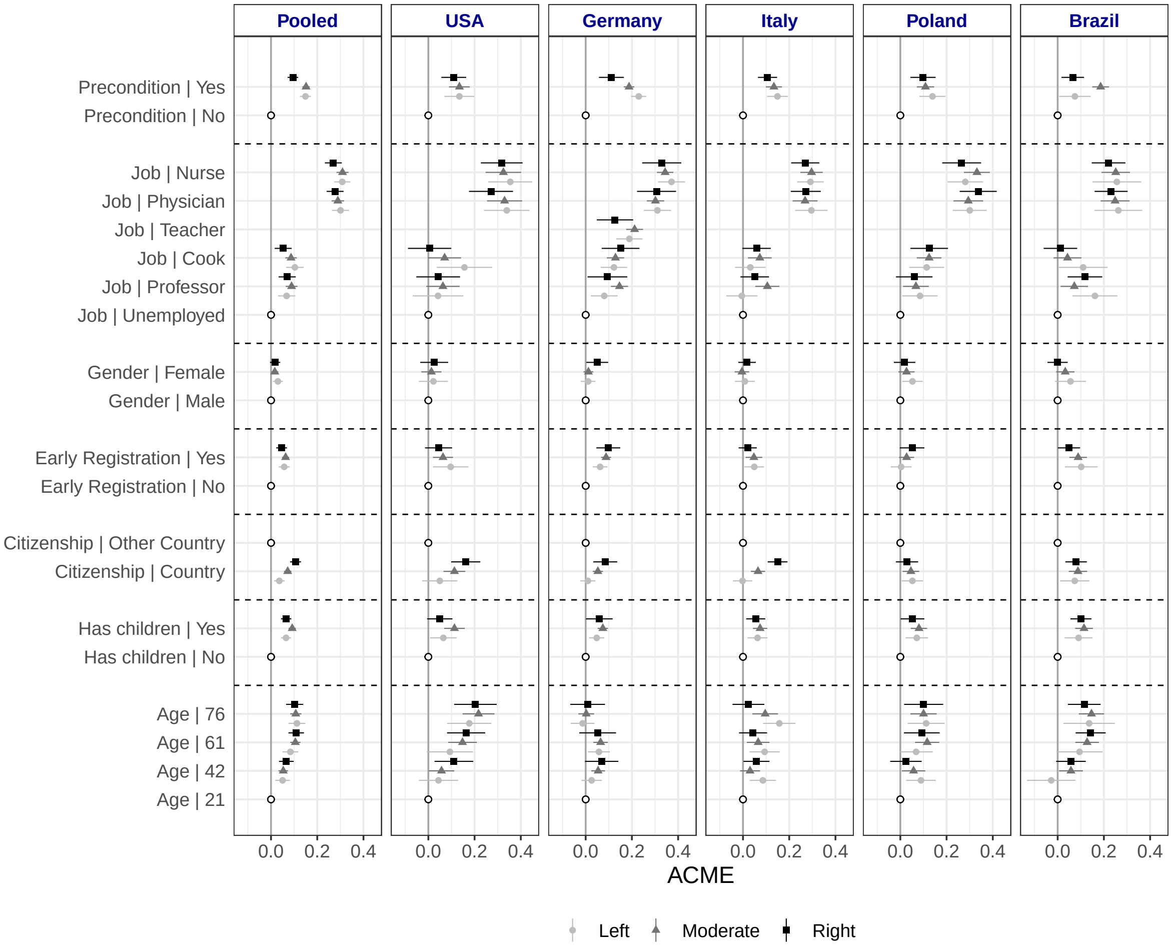

Supplementary Figure 11: Average Marginal Component effect of patient's citizenship status conditional on respondent's political ideology

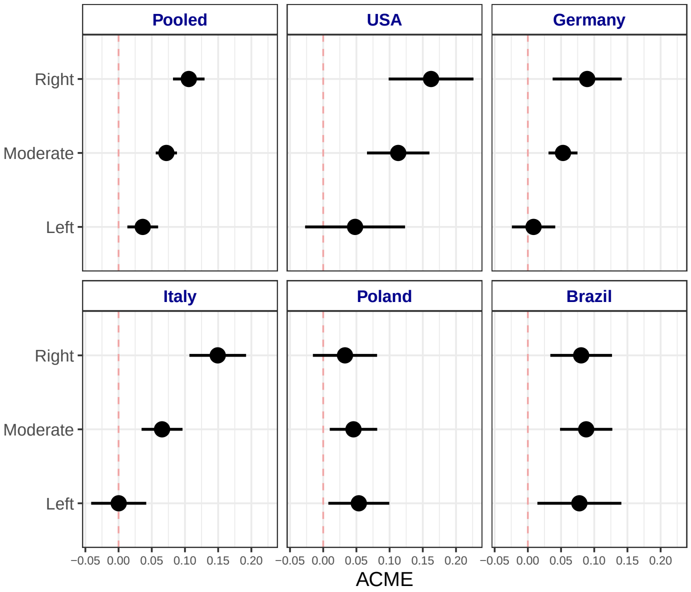

Supplementary Figure 12: Average Marginal Component effect of patient's COVID precondition status conditional on respondent's precondition status

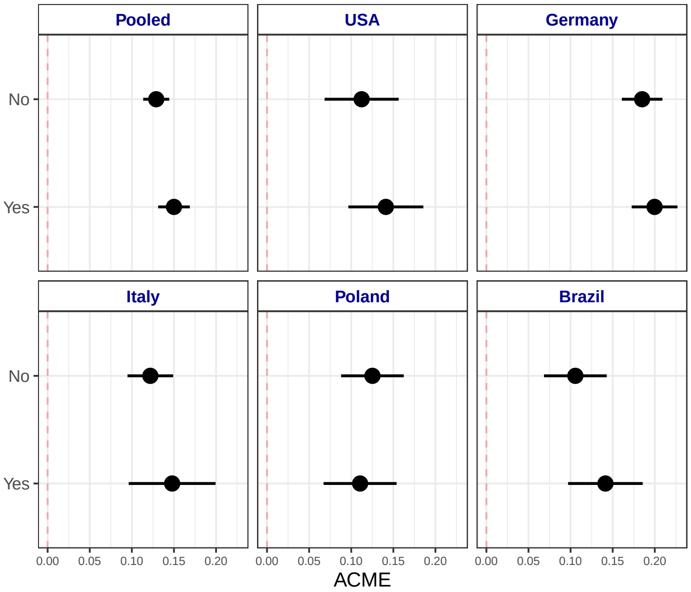

Supplementary Figure 13: Average Marginal Component effect of a patient having children conditional on respondent's being a parent

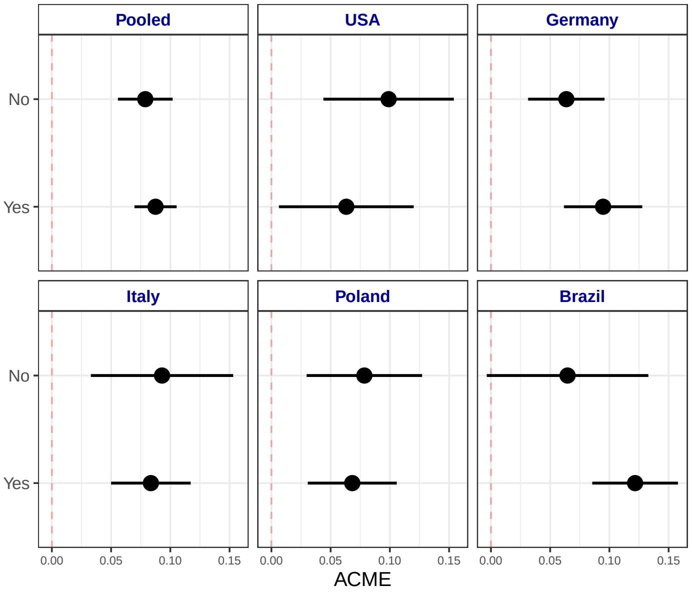

Supplementary Figure 14: Average Marginal Component effect of a patient's sex conditional on respondent's sex

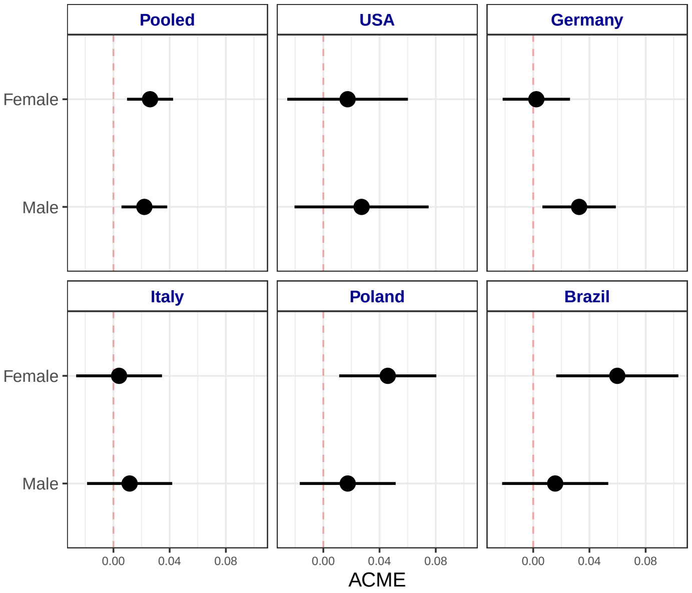

Supplementary Table 3: Estimated loadings from two-factor model for institutional trust.

Trust in...	Factor 1	Factor 2
Federal (National) Government	0.91	0.13
State (Regional) Government	0.75	0.40
Scientists	0.15	0.88
Media	0.31	0.71
Healthcare system	0.57	0.54

3.4 Regression Output

Supplementary Table 4: Average component marginal effects and 95% confidence intervals for prioritizing individuals in the five country and combined samples.

	Pooled	USA	Germany	Italy	Poland	Brazil
(Intercept)	0.10	0.04	0.09	0.16	0.13	0.08
	[0.08; 0.12]	[−0.02; 0.09]	[0.06; 0.12]	[0.12; 0.19]	[0.08; 0.17]	[0.04; 0.12]
Age42	0.05	0.06	0.05	0.05	0.06	0.04
	[0.04; 0.07]	[0.02; 0.11]	[0.02; 0.07]	[0.02; 0.08]	[0.02; 0.09]	[−0.00; 0.08]
Age61	0.10	0.14	0.06	0.07	0.10	0.13
	[0.09; 0.12]	[0.10; 0.19]	[0.04; 0.09]	[0.04; 0.10]	[0.06; 0.14]	[0.09; 0.17]
Age76	0.11	0.20	−0.00	0.09	0.11	0.13
	[0.09; 0.13]	[0.15; 0.25]	[−0.03; 0.03]	[0.05; 0.13]	[0.06; 0.15]	[0.09; 0.17]
EarlyRegistrationYes	0.06	0.07	0.09	0.04	0.03	0.08
	[0.05; 0.07]	[0.03; 0.10]	[0.07; 0.10]	[0.01; 0.06]	[0.00; 0.05]	[0.05; 0.11]
GenderFemale	0.02	0.02	0.02	0.01	0.03	0.04
	[0.01; 0.04]	[−0.01; 0.06]	[0.00; 0.04]	[−0.01; 0.03]	[0.01; 0.06]	[0.01; 0.07]
HasChildrenYes	0.08	0.09	0.06	0.07	0.07	0.11
	[0.07; 0.09]	[0.06; 0.12]	[0.05; 0.08]	[0.04; 0.09]	[0.04; 0.09]	[0.08; 0.14]
JobCook	0.08	0.07	0.13	0.06	0.12	0.05
	[0.06; 0.10]	[0.01; 0.12]	[0.10; 0.16]	[0.02; 0.09]	[0.08; 0.16]	[0.00; 0.09]
JobProfessor	0.08	0.06	0.12	0.06	0.07	0.09
	[0.06; 0.10]	[0.01; 0.11]	[0.09; 0.15]	[0.02; 0.10]	[0.03; 0.11]	[0.05; 0.14]
JobTeacher	0.16		0.20			
	[0.14; 0.19]		[0.17; 0.23]			
JobPhysician	0.29	0.32	0.31	0.28	0.31	0.25
	[0.27; 0.31]	[0.27; 0.37]	[0.27; 0.34]	[0.24; 0.31]	[0.26; 0.35]	[0.20; 0.29]
JobNurse	0.30	0.33	0.35	0.28	0.30	0.24
	[0.28; 0.32]	[0.28; 0.38]	[0.32; 0.38]	[0.25; 0.32]	[0.26; 0.34]	[0.19; 0.28]
PreconditionYes	0.14	0.12	0.19	0.13	0.11	0.13
	[0.12; 0.15]	[0.09; 0.15]	[0.17; 0.21]	[0.10; 0.15]	[0.09; 0.14]	[0.10; 0.16]
CitizenshipCountry	0.07	0.11	0.04	0.07	0.05	0.08
	[0.06; 0.08]	[0.08; 0.15]	[0.02; 0.06]	[0.05; 0.09]	[0.02; 0.07]	[0.06; 0.11]
R^2	0.10	0.14	0.11	0.09	0.09	0.10
Adj. R^2	0.10	0.14	0.10	0.09	0.09	0.09
Num. obs.	36936	3824	13218	8016	6302	5576
N Clusters	4354	436	1578	952	743	645

4 Priority Ranking Task

Supplementary Figure 15: Distribution of priority groups chosen in each rank for the pooled sample

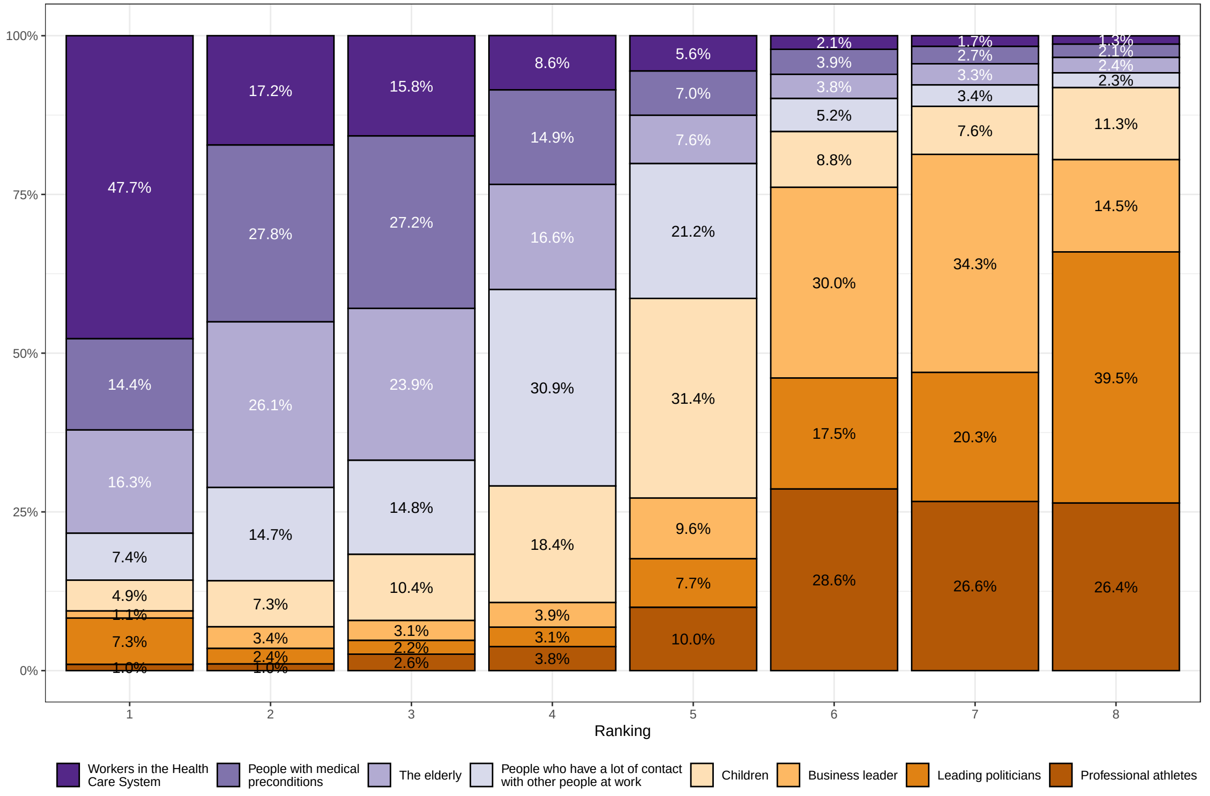

Note: The numbers inside the stacked bars reflect the percentage of times that respondents chose the individual group in a ranking—for instance, *workers in the health care system* were positioned in the first rank in 47.7% of the instances.

Supplementary Figure 16: Distribution of rankings for each priority group

(a) Pooled

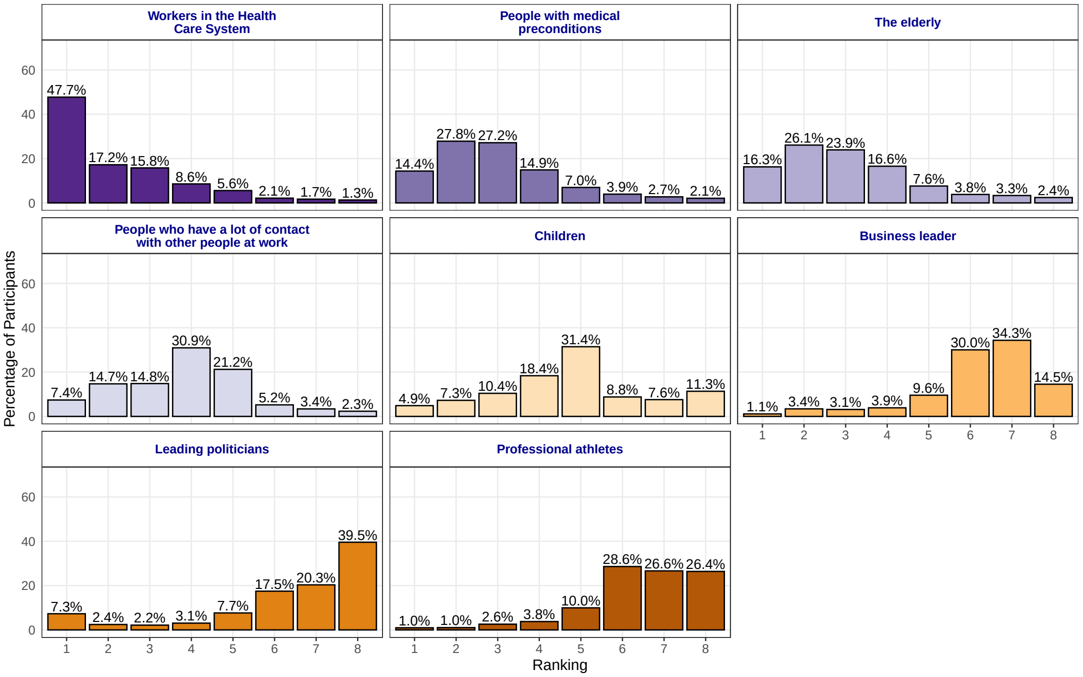

(b) USA

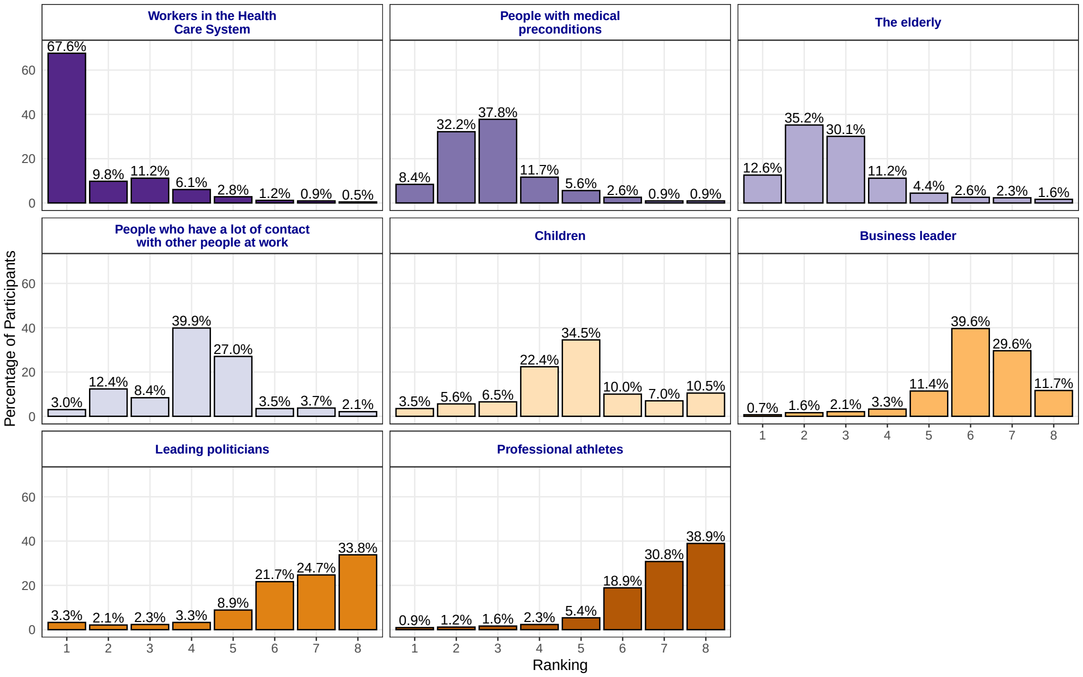

Supplementary Figure 16: Distribution of rankings for each priority group

(c) Germany

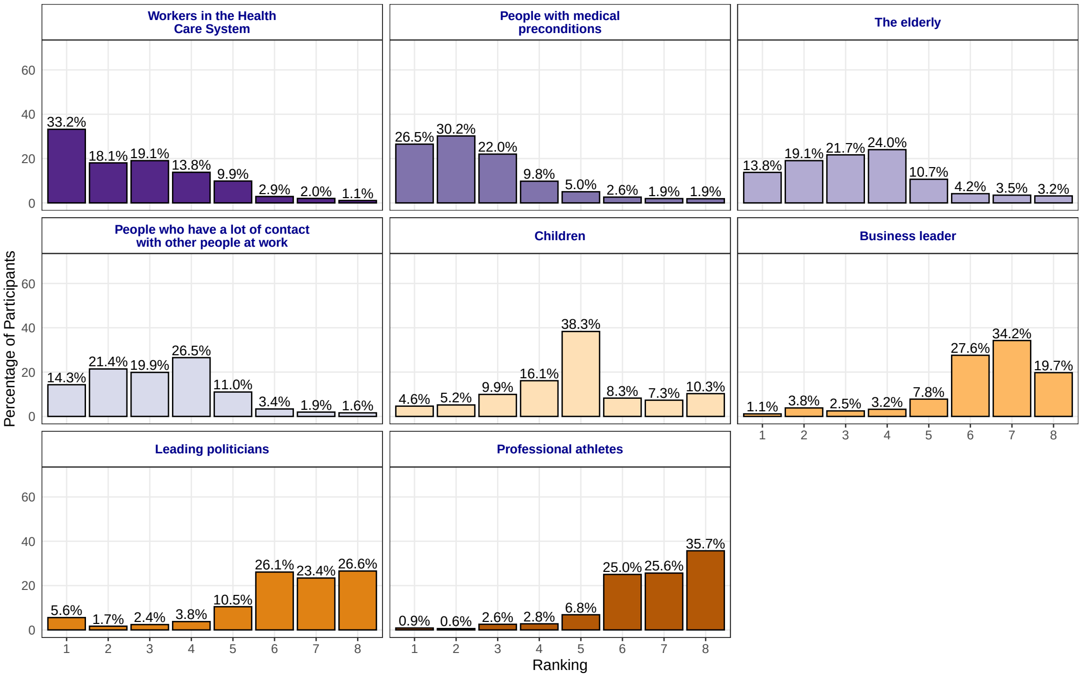

(d) Italy

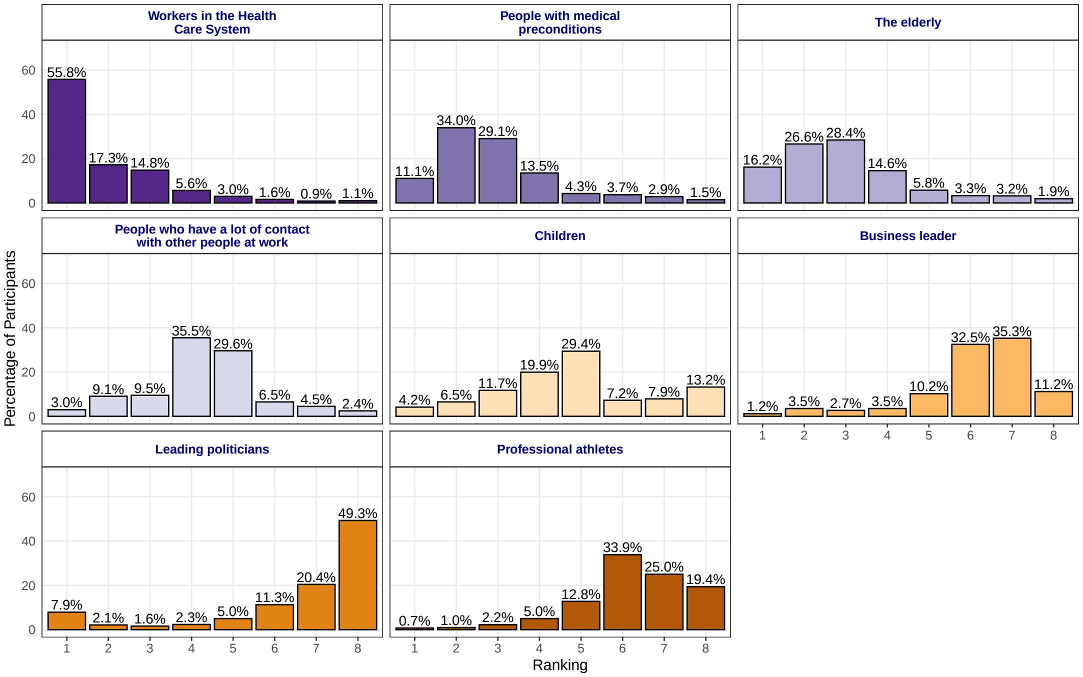

Supplementary Figure 16: Distribution of rankings for each priority group

(e) Poland

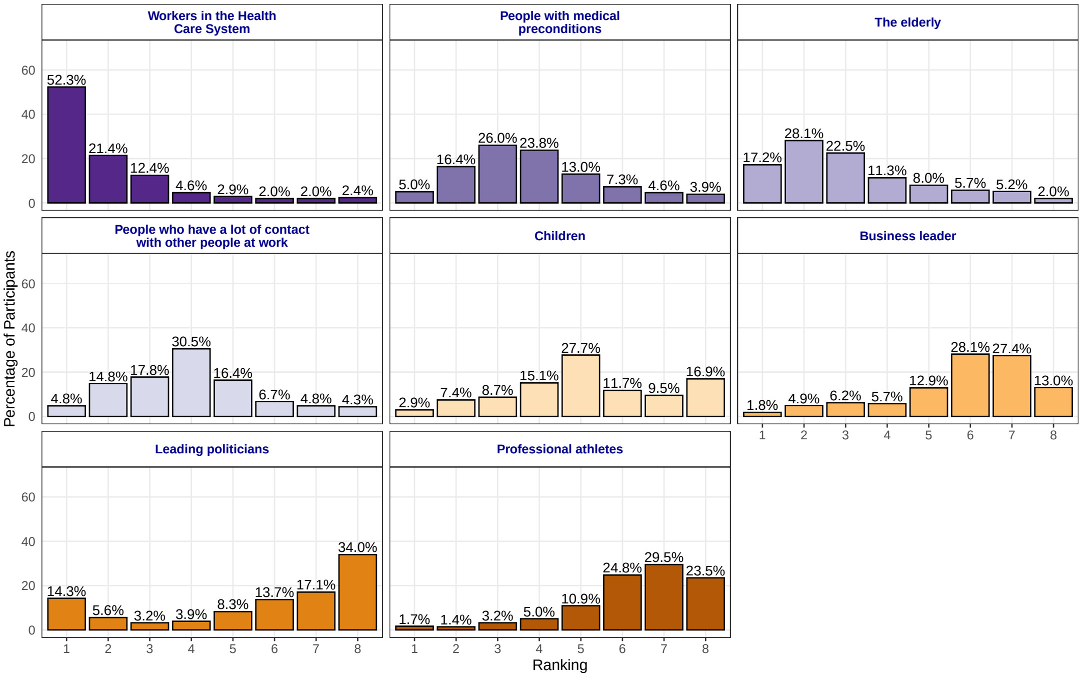

(f) Brazil

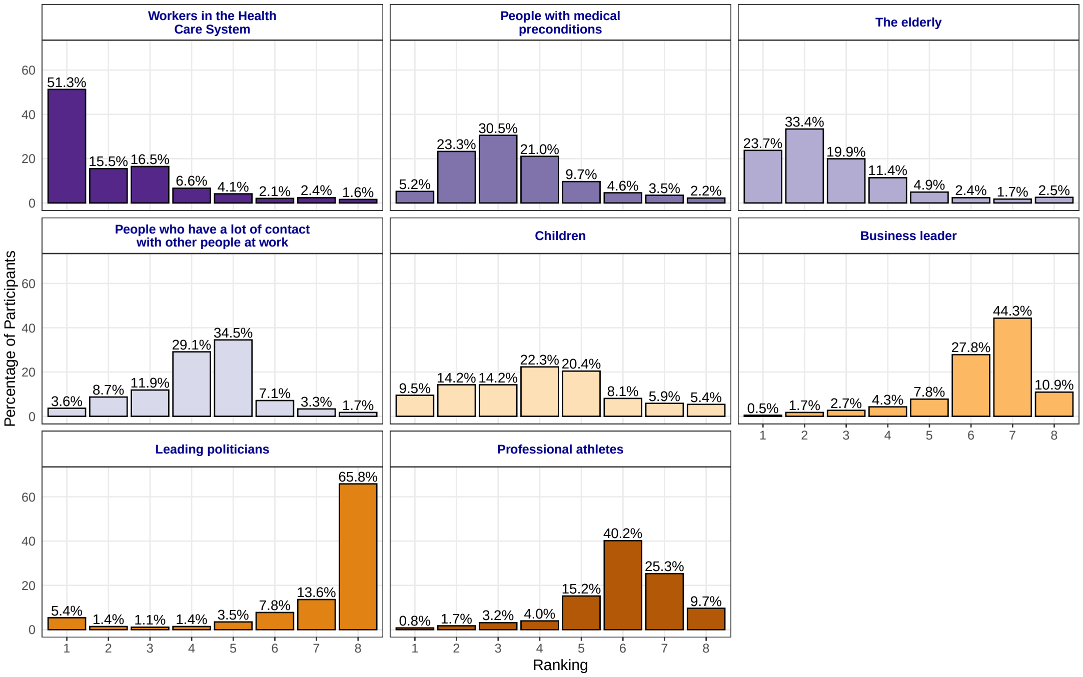

Supplement: Supplementary file 1 — Supplementary Information [file 41599_2022_1392_MOESM1_ESM.pdf]
